# Liver Enzymes in Children with beta-Thalassemia Major: Correlation with Iron Overload and Viral Hepatitis

**DOI:** 10.3889/oamjms.2015.059

**Published:** 2015-05-28

**Authors:** Khaled M. Salama, Ola M. Ibrahim, Ahmed M. Kaddah, Samia Boseila, Leila Abu Ismail, May M. Abdel Hamid

**Affiliations:** 1 *Faculty of Medicine, Cairo University, Cairo, Egypt*; 2 *National Research Center, Child Health Department, El Buhouth st., Dokki, Cairo 12311, Egypt*

**Keywords:** Beta thalassemia, egyptian children, viral hepatitis, iron overload, liver enzymes

## Abstract

**BACKGROUND:**

Beta Thalassemia is the most common chronic hemolytic anemia in Egypt (85.1%) with an estimated carrier rate of 9-10.2%. Injury to the liver, whether acute or chronic, eventually results in an increase in serum concentrations of Alanine transaminase (ALT) and Aspartate transaminase (AST).

**AIM:**

Evaluating the potentiating effect of iron overload & viral hepatitis infection on the liver enzymes.

**PATIENTS AND METHODS:**

Eighty (80) thalassemia major patients were studied with respect to liver enzymes, ferritin, transferrin saturation, HBsAg, anti-HCV antibody and HCV-PCR for anti-HCV positive patients.

**RESULTS:**

Fifty % of the patients were anti-HCV positive and 55% of them were HCV-PCR positive. Patients with elevated ALT and AST levels had significantly higher mean serum ferritin than those with normal levels. Anti-HCV positive patients had higher mean serum ferritin, serum ALT, AST and GGT levels and higher age and duration of blood transfusion than the negative group. HCV-PCR positive patients had higher mean serum ferritin and serum ALT and also higher age and duration of blood transfusion than the negative group.

**CONCLUSION:**

Iron overload is a main leading cause of elevated liver enzymes, and presence of HCV infection is significantly related to the increased iron overload.

## Introduction

Thalassemia is among the most common genetic disorders, and nearly 7% of the world population carry a hemoglobinopathy [1]. The burden of this disorder in many regions is of such a magnitude that it represents a major public health concern [2]. Transfusion therapy which is the main way of treatment allows for normal growth and suppresses ineffective erythropoiesis [3]. Iron overload is often inevitable, especially when iron chelating agents are not used properly [4]. Even in the absence of transfusion, the accelerated rate of erythropoiesis enhances dietary iron absorption from the gut, resulting in a chronic state of iron overload [5]. As patients with thalassemia commonly receive transfusions, they are exposed to transfusion-associated infections, where hepatitis B and hepatitis C are the most common infections detected [6]. Although the risk of post-transfusion hepatitis C virus (HCV) infection dropped significantly after the national screening of blood in 1993, more than 20% of children who were multitransfused after that date were HCV-RNA positive [7]. In Egypt, prevalence rate of HCV antibodies seropositivity in thalassemic children at 2011was 51.7% [8] while in the study of El-Faramawy et al., (2012) [9], a prevalence of 48 % was reported.

Iron overload and hepatitis-C virus (HCV) infection, have been implicated in the evolution of liver disease, in patients with transfusion-dependent beta-thalassaemia major (BTM). The impact of these factors in late stages of liver disease with BTM, has not been extensively studied yet.

Interrelationship between iron overload, HCV infection and liver injury is still controversial. Multicenter cross-sectional studies have reported that the development and the severity of liver injury are strongly related to the extent of liver iron overload and to the presence of chronic HCV infection [10, 11].

Long-term observation of thalassemics who had undergone bone marrow transplantation showed that severe iron overload and chronic HCV infection were independent risk factors for liver injury [12]. Hepatitis virus C infection is the main risk factor for liver injury in transfusion-dependent thalassemics [13]. Dimitrios et al (2013) [14] on the other hand suggested that in the late stages of liver disease in BTM patients, iron overload may be the critical determinant, since fibrosis is related to the minimal haemosiderosis, independently of HCV history. Injury to the liver, whether acute or chronic, eventually results in an increase in serum concentrations of Alanine transaminase (ALT) and Aspartate transaminase (AST) [15].

We aimed in this study to determine the relationship between liver enzymes with serum iron status and HCV infection in thalassemic patients and to throw light on the percentage of HBV and HCV in the studied patients.

## Subjects and Methods

This cross sectional study was conducted on 80 patients with beta-thalassemia major. They were recruited from the Hematology Clinic in New Children Hospital, Cairo University, their ages ranged from 5 to 18 years with a mean age of 11.09 ± 4.156 years. Forty three (53.7%) of them were males and thirty seven (46.3%) were females. An informed parental consent was obtained from every case before the study. Inclusion criteria of patients were; age ranging from 5 to 18 years, undergoing regular **blood** transfusion and had clinical follow up for at least 24 months before study. Any child with a history of liver affection (congenital, autoimmune hepatitis, hereditary hemochromatosis) was excluded from the study. All subjects underwent the following: I - Complete history taking: A questionnaire was planned to fulfil: 1) Demographic data: name, age, sex, socioeconomic classes, consanguinity and positive family history of thalassemia. 2) Medical history: age of onset duration, age of start of blood transfusion, and its frequency, history of splenectomy and history of liver affection. 3) Regimen of management: type of therapy received, including chelation therapy (dose, age of start, complications and compliance). II - Complete physical and clinical examination: Thorough clinical examination with particular emphasis on presence of pallor, jaundice, and signs of thalassemic features. Abdominal examination for hepatosplenomegally. III - Laboratory investigations: Blood was collected by venipuncture and was allowed to clot naturally and completely. The serum was separated from the clot; hemolysis of the RBC was avoided. Care was taken to ensure that the serum samples are not contaminated. Collection tubes were iron free. Serum stored in Stopper tubes at 2-8°C till time laboratory investigations which included: 1) Iron status as indicated by serum ferritin level and transferrin saturation. 2) Liver functions as indicated by serum level of Alanine transaminase (ALT), Aspartate transaminase (AST) and Gamma-glutamyl transpeptidase (GGT). 3) Detection of hepatitis viral infection by Hepatitis B surface antigen (HBsAg), Anti-hepatitis C virus (anti-HCV) antibody and RNA-PCR testing for HCV positive patients.

### Serum ferritin level

Diametra, CA06034, Italy, Direct immunoenzymatic determination of ferritin in human serum or plasma [16]. Expected values: Normal range: male 6-180 ng/L female 20-400 ng/L. Transferrin saturation: It is the ratio of serum iron and total iron-binding capacity, multiplied by 100 (normal range: 15-50%). Iron and Total Iron Binding Capacity (TIBC) were measured by CA0370, USA, Quantitative colorimetric determination of iron and unsaturated iron-binding capacity in serum [17]. Serum iron expected values in children are 40-120 μg/dL. TIBC expected values in children are 250-400 μg/dL. Alanine transaminase (ALT): Stanbio ALT/GPT (UV-Rate), CA0930, USA, Quantitative determination of ALT/GPT in serum [18]. Expected values: Normal range: 0-38 U/L at 37°C Aspartate transaminase (AST): Stanbio AST/GOT (UV-Rate), CA0920, USA, Quantitative determination of AST/GOT in serum [19]. Normal range: 0-40 U/L at 37°C. Gamma-glutamyl transpeptidase [20] (GGT): Centronic GmbH, CA85456, Germany, Enzymatic colorimetric test for the determination of L-γ-glutamyltransferase (Szasz method) in serum and plasma. Expected values: normal range: male 11-49 IU/L, female 7-32 IU/L. Hepatitis B surface antigen (HBsAg): Prechek Bio, Inc. CA92806, USA, Diagnostic kit for hepatitis B virus surface antigen (ELISA), one-step incubation, double-antibody Sandwich principale [21]. Anti-hepatitis C virus (anti-HCV) antibody: Prechek Bio, Inc. CA92806, USA, Diagnostic kit for antibody to hepatitis C virus (ELISA), two-step incubation, indirect principale [22]. HCV-PCR: Positive cases for HCV antibodies were subjected to PCR for HCV-RNA reverse transcriptase PCR (RT-PCR). Qualitative estimation of HCV-RNA in serum was detected by nested RT-PCR assay.

### Statistical analysis

Data were analyzed using the statistical package for social science (SPSS). Computer software package SPSS 15.0 was used in the analysis. For quantitative variables, mean, standard deviation, minimum, and maximum (as measures of variability) were presented. Frequency and percentages were presented for qualitative variable. ANOVA, Independent T, Mann-Whitney and Kruskal Wallis tests were used to estimate differences in quantitative variables. Chi-square and test was used to estimate differences in qualitative variables. Spearman’s rank correlation test was used to determine the relationship between different numerical variables. For all tests, probability values (P) of less than 0.05 were regarded as statistically significant.

## Results

This study included 80 patients with beta thalassemia major; their age ranged from 4-18 years with a mean age of 11.09 ± 4.156 years. They were 43 males (53.75%) and 37 (46.25%) females. Positive consanguinity was present in 43 (53.75%) patients and 35 (43.75%) had positive family history of other members affected. Thirty two patients (40%) suffered from jaundice and 34 (42.5%) had thalassemic features. All patients were on regular blood transfusion with a mean frequency of 12.94 ± 4.31 times per year. The age of onset of blood transfusion ranged from 2-12 month with a mean of 6.84 ± 2.9 months. There was a significant decrease in the frequency of blood transfusion per year in patients who underwent splenectomy (n = 55), compared to the pre-splenectomy period (P = 0.034).

Seventy five patients were on regular chelation therapy; 78.7 % of these patients were compliant with chelators, 5 patients only were not on any chelation therapy because of the complication of oral chelators or refusal of injections. All the thalassemic patients included in the study were on folic acid, calcium and vitamin D therapy. Only 47.5 % of the patients received L-carnitine. No significant difference was noted in the mean level of ferritin, transferritin saturation or number of blood transfusion per year between patients on L-carnitine and those who did not receive it (data not shown).

In comparison of biochemical parameters in the two types of iron chelators, only serum ALT showed a significant decrease in patients on deferiprone therapy (p=0.013) (Table 1).

**Table 1:** Comparison between deferiprone and deferoxamine as regards the biochemical parameters

Parameter	Deferiprone (mean ± SD)	Deferoxamine (mean ± SD)	p-value
Serum ALT U/L	95.21 ± 74.070	158.44 ± 135.207	0.013*
Serum AST U/L	59.04 ± 95.98	81.72 ± 98.19	0.39
Serum GGT IU/L	23.83 ± 52.24	25.25 ± 22.97	0.91
Serum ferritin ug/L	805.61 ± 424.38	904.61 ± 374.32	0.38
TSAT %	76.46 ± 39.25	72.44 ± 27.03	0.68

* p value is significant if < 0.05

None of our patients was positive for HBSAg; however 40 (50%) patients were anti-HCV positive, where only 22 of them (55%) were PCR positive which contributes to 27.5% of the 80 patients included in the study.

**Figure 1:**
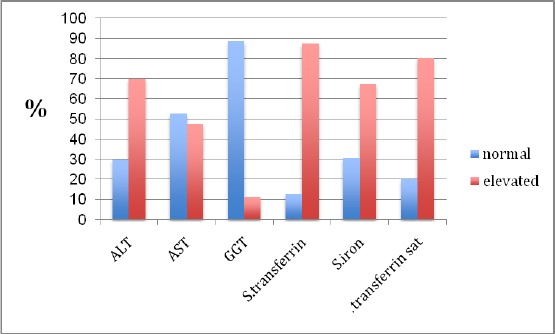
*Percentage of patients with normal and elevated levels of biochemical parameters*.

According to the level of each biochemical parameter the patients were divided into 2 groups, one with normal level and the other with high level. The percentage of patients with elevated levels of ALT and parameters of iron status are very high (Fig. 1).

Mean serum ferritin level was significantly higher in patients with either elevated ALT level or elevated AST level as compared to those with normal ALT or AST levels (p=0.007 and 0.004 respectively) (Table 2).

**Table 2:** The mean ferritin level as regard to ALT and AST level

	Ferritin (ng/L) Mean ± SD	P value
Pts.with normal ALT level (N=24)	633.96 ± 360.1	
Pts. with high ALT level (N=56)	903.21 ± 412.47	0.007*
Pts. with normal AST level (N=42)	698.74 ± 371.8	
Pts. with high AST level (N=38)	959.16 ± 420.33	0.004*

* p value is significant if < 0.05

A significant high number of patients with +ve anti –HCV antibody had an above normal level of either ALT or AST (Table 3).

**Table 3:** Comparison between normal and high ALT level as regard to the number of patients with positive infection of HCV

	Anti-HCV		P value

	+ve (40)	-ve (40)	
Pts.with normal ALT level (N=24)	7	17	0.027*

Pts. with high ALT level (N=56)	33	23

Pts. with normal AST level (N=42)	13	29	0.001*

Pts. with high AST level (N=38)	27	11	

* p value is significant if < 0.05

In addition, by comparing the mean level of liver enzymes (ALT, AST and GGT), ferritin and transferritin saturation in patients with +ve Anti-HCV to those with –ve Anti-HCV, there were significantly higher levels of all parameters (except for TSAT%) in patients with +ve Anti-HCV. Serum ALT and ferritin on the other hand are the only parameters that showed significantly higher levels in the +ve HCV-PCR patients as compared to those with –ve HCV-PCR (p= 0.007and 0.001 respectively (Table 4).

**Table 4:** The mean ± SD of liver enzymes, ferritin and transferritin saturation regarding Anti-HCV and HCV-PCR results

	Anti-HCV *(N=80)*			HCV-PCR *(N=40)*		
	+ve	- ve	p-value	+ve	- ve	p-value
ALT (U/L) Mean ± SD	137.2 ±106.8	80.2 ± 68.8	0.006*	153.86 ± 115.89	91.47 ± 78.57	0.007*
AST (U/L) Mean ± SD	87 ± 123.4	38.3 ± 36.2	0.019*	82.00 ±86.89	55.36±95.77	0.259
GGT (U/L) Mean ± SD	34.3 ± 13.17	62.25 ±8.5	0.037*	30.15 ± 30.44	21.31 ±49.96	0.44
Ferritin (μg /L) Mean ± SD	933.82 ± 443.55	711.05 ±354.19	0.015*	1060.46 ± 460.32	732.16 ±360.04	0.001*
Transferrin Saturation % Mean ± SD	68.0 ±22.77	80.45 ±44.82	0.122	68.64 ±23.43	75.47 ±40.33	0.458

* P value is significant if < 0.05

The mean level of ALT and GGT in patients with high level of ferritin (> 320 μg/l) are significantly higher as compared to those with ferritin level (< 320 μg/l), (p = 0.04 and 0.05 respectively) (Table 5). No such difference was found on the other hand, when comparing the mean levels of liver enzymes (ALT, AST, GGT), in patients with high level of transferritin saturation to those with normal level.

**Table 5:** The mean ± SD of ALT, AST and GGT according to Ferrintin level

Parameter	Ferrintin level (>320 ug/l) n=63	Ferrintin level (<320 ug/l) n=17	P
ALT (U/L) Mean ± SD	118.27 ± 97.03	72.88 ± 72.77	0.04^*^
AST (U/L) Mean ± SD	59.58 ± 63.66	74.18 ± 165.55	N.S
GGT (IU/L) Mean ± SD	26.53 ± 50.66	13.39 ± 9.56	0.05^*^

* p value is significant if < 0.05

**Figure 2:**
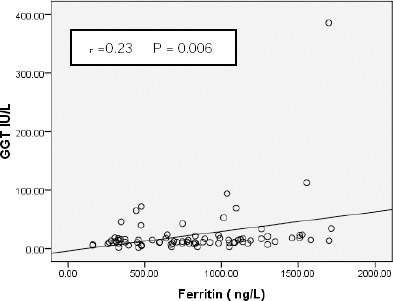
*Correlation between serum ferritin and GGT level*.

Serum ferritin level correlated positively with GGT level (r = 0.23 and p = 0.006) as shown in Fig. 2, but not with ALT or AST levels.

Serum ALT level correlated positively with the age of the patients (r = 0.3 and p = 0.01), and with the duration of blood transfusion (r = 0.3 and p = 0.01). No correlation on the other hand was found between transferritin saturation and either age of patients or duration of blood transfusion.

## Discussion

The aim of this study was to evaluate the potentiating effect of iron overload and viral hepatitis infection on liver enzymes as a marker for hepatic injury in Egyptian children with thalassemia major, and to throw light on the percentage of HBV and HCV infection in studied thalassemic children.

This study was conducted on 80 thalassemic patients, 55 of them underwent splenectomy which resulted in a significant decrease in the mean number of times of blood transfusion per year compared to the pre-splenectomy period (p = 0.034). These results are in accordance with the study of Al-Salem and Nasserulla (2002) [23] which showed that in children who had total splenectomy, their postsplenectomy transfusion requirements decreased from a preoperative mean of 17.8 transfusions per year to a postoperative mean of 10 transfusions per year. In addition, Morsy et al., (2008) [24] revealed that the total amount of blood given over a whole year was significantly lower in the splenectomized group of their patients.

Our results showed that only 7 patients (12.2%) had complications due to deferiprone therapy in the form of elevated liver enzymes, arthralgia and GIT upsets in the form of abdominal pain, while there were no reported significant complications from deferoxamine. Same observation was noted in other previous studies [25, 26].

In the present study no difference was noted in the efficacy of the two iron chelators as denoted by absence of significant differences in serum levels of iron parameter between patients receiving deferiprone and those receiving deferoxamine. Previous comparative studies have shown that at comparable doses the efficacy of deferiprone(DFP) in removing body iron is similar to that of desferoxamine (DFO) and deferiprone seems to be a valuable alternative and an effective iron chelator for patients unable or unwilling to use deferoxamine [25, 27]. El-Alfy et al., (2010) [28], on the other hand revealed that deferiprone use was associated with a high significant decline in mean serum ferritin level (P < 0.0005).

Our results showed higher levels of liver enzymes in patients treated by deferoxamine but the difference was significant only in serum ALT level.

Thalassemia major patients receiving L-carnitine therapy need significantly less blood transfusions per year [29], and there is an improvement in their serum ferritin level [30]. L-carnitine intake by 47.5% of our thalassemic patients had no significant effect on their serum ferritin level, transferrin saturation or number of blood transfusions per year. This could be due to inadequate intake of L- carnitine due to irregularity of receiving it or the duration of intake was too short to improve the condition.

The mean serum ferritin level was significantly higher in thalassemic patients with elevated ALT or elevated AST compared to those patients with normal levels (p = 0.007 and 0.004 respectively). Furthermore, the mean ALT and GGT levels were significantly higher in patients with high ferritin level as compared to those of normal ferritin level. These findings are in agreement with other previous studies [3, 31] who reported that abnormal ALT level is associated with higher ferritin and transferring saturation.

Fortunately none of the patients included in this study was HBSAg positive. Absence of positive HBSAg among studied thalassemic patients was also reported by Amli et al., (2008) [3] and Omar et al., (2011) [8]. Hepatitis B virus infection occurred in only one patient (2%) out of 50 thalassemics in the study of El Gawhary et al., (2009) [32], While in the study of El-Faramawy et al., (2012) [9] 12% of patients were infected with HBV. The decrease in the prevalence of hepatitis B infection in this study and some other studies reflects the efficacy of the vaccination program and effective screening of blood and blood products for these patients. Although the prevalence of HBsAg among Egyptian polytransfused children decreased, it is still high in some studies in different geographical areas, and this could be attributed to the high infection rates among those children despite vaccination against HBV, as they might not have responded adequately to vaccination or may not be fully vaccinated.

Fifty percent of our studied patients were anti-HCV positive and only 55% of these positive patients were HCV-PCR positive which contribute to 27.5% of the 80 patients included in the study.

Similar results were reported in the study of Omar et al (2011) [8], as 51.7% of their patients were HCV antibodies positive and only 25% of the studied patients were positive for HCV infection by PCR. In addition, the study of Din et al, (2014) [33] revealed that 49% of their thalassemic patients were positive for anti-HCV antibodies.

Lower percentage of thalassemic patients with HCV positive antibodies (40.5%) were reported in the study of Masour et al., (2012) [34]. Furthermore, Hussein (2014) [35], reported that 48 of 200 (24%) of multi-transfused Egyptian children with thalassemia were positive for HCV antibodies.

Higher results were obtained in the study of Elalfy et al (2010) [28] who revealed that 82% of the thalassemic patients were HCV antibody positive and 49% of them were viremic (HCV RNA positive). A study in Tanta University Hospital also reported that 40% of the multi-transfused children had positive PCR for HCV [36].

In our study, anti-HCV positive patients had higher mean serum ferritin, serum ALT, AST and GGT levels than the negative group (p = 0.015, 0.006, 0.019 and 0.037 respectively). Also, HCV-PCR positive patients had higher mean serum ferritin and serum ALT than the negative group (p=0.001 and p=0.007 respectively).

The findings of Ocak et al., (2006) [37] indicated that the patients who were anti-HCV positive had a significantly higher peak serum ALT level than anti-HCV-negative patients. Ameli et al., (2008) [3], found that serum iron was significantly higher in anti-HCV positive patients compared to the negative group, and the same with the PCR positive group. Moreover, Omar et al., (2011) [8] stated that HCV antibody positivity was significantly higher with higher serum ferritin and higher liver transaminases and that PCR positivity was significantly related to patients’ serum ferritin level, which showed statistically significant positive correlation with liver transaminases.

The results of this work showed that the level of serum ALT was highly significantly correlated with age of the patients and duration of blood transfusion (p=0.01 and 0.01 respectively).

It was concluded that iron overload is a main leading cause of elevated liver enzymes and presence of HCV infection is significantly related to the increased iron overload. Although the risk of transfusion-associated hepatitis has been reduced, it is still high.
